# Myocardial iron assessment by T1 cardiovascular magnetic resonance at 1.5 Tesla

**DOI:** 10.1186/1532-429X-16-S1-P295

**Published:** 2014-01-16

**Authors:** Mohammed H Alam, Taigang He, Gillian C Smith, Arun J Baksi, Ricardo Wage, Peter Drivas, Yanqiu Feng, David Firmin, Dudley J Pennell

**Affiliations:** 1 NIHR Cardiovascular Biomedical Research Unit, Royal Brompton Hospital, London, UK; 2 Imperial College London, London, UK; 3 School of Biomedical Engineering, Southern Medical University, Guangzhou, China

## Background

Heart failure secondary to cardiac siderosis is the prevalent cause of mortality in patients with primary or secondary iron overload. Myocardial iron assessment using T2* has been successfully calibrated against myocardial iron at 1.5T and the black blood (BB) technique has demonstrated high reproducibility such that it has become the clinical gold standard. T1 appears to correlate with T2* in cases of significant iron loading (T2* < 20 ms) but this relationship weakens when T2* is in the normal range. We evaluated this further.

## Methods

69 subjects (40 male, aged 14 to 81 years) comprising 20 healthy volunteers (controls) and 49 patients (thalassemia major 22, sickle cell disease 9, hereditary hemochromatosis 8, other iron overload conditions 10) referred for routine iron assessment were recruited. The same mid-ventricular short axis cardiac slice was used to acquire both BB T2* images and to generate T1 MOLLI maps for each subject at 1.5T (Avanto, Siemens AG Healthcare Sector, Erlangen, Germany). 20 subjects underwent repeat studies on the same day to evaluate reproducibility. Regions of interest were selected in the septum using CMRtools.

## Results

**Figure 1:**
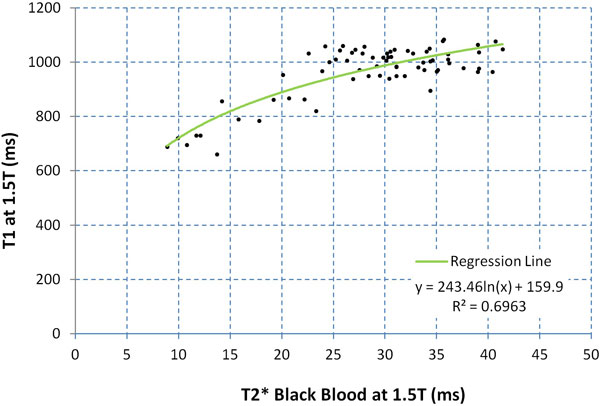
Scatter plot showing relationship between T1 and T2* at 1.5T.

There was a reasonable correlation between BB T2* and T1 values described by a logarithmic relationship (R^2 ^= 0.696; Figure 1). In the control group, all of whom had normal T2* values ( > 20 ms), mean T1 was 1003 ± 71 ms, thus defining the lower limit of normal as 932 ms. All patients with T2* < 20 ms (n = 10) had a low T1 value (mean 752 ± 59 ms). Of the patients with T2* > 20 ms (n = 39), T1 was normal in 90% (n = 35). Four patients with thalassemia had T2* indicating mild but non-clinically significant iron loading (range 20.7-34.4 ms), and their T1 values were likewise below normal (821-896 ms). There was excellent intra-observer, inter-observer and inter-study reproducibility of T1 measurement, with coefficients of variation (CoV) of 0.56%, 0.98% and 1.8%.

## Conclusions

In this analysis, T1 mapping using a MOLLI sequence identified all those individuals with significant iron loading as defined by the current gold standard T2* technique and has excellent reproducibility. As expected, T1 can also identify patients with mild cardiac iron loading that is not considered clinically significant. It is possible that T1 may have a clinical role in non-cardiac conditions with very low levels of iron loading, where factors other than iron affect the T2* signal, however tissue calibration remains lacking at this time. Further work is needed to determine whether there might be clinically significant advantages of T1 over the T2* cardiac technique at 1.5T for assessment of cardiac iron, but there are none at this time.

## Funding

This research was supported by the NIHR Cardiovascular Biomedical Research Unit at Royal Brompton & Harefield NHS Foundation Trust and Imperial College London.

